# Monetary Cost of the MyPlate Diet in Young Adults: Higher Expenses Associated with Increased Fruit and Vegetable Consumption

**DOI:** 10.1155/2019/2790963

**Published:** 2019-05-02

**Authors:** Rashel L. Clark, Oluremi A. Famodu, Makenzie L. Barr, Rebecca L. Hagedorn, Jane Ruseski, Jade A. White, Caitlin M. Warner, Alexandra M. Morrell, Pamela J. Murray, I. Mark Olfert, Joseph W. McFadden, Marianne T. Downes, Sarah E. Colby, Melissa D. Olfert

**Affiliations:** ^1^West Virginia University, Davis College of Agriculture, Natural Resources, and Design, Division of Animal and Nutritional Sciences, Morgantown, WV, USA; ^2^West Virginia University, College of Business and Economics, Morgantown, WV, USA; ^3^West Virginia University, School of Medicine Morgantown, WV, USA; ^4^University of Tennessee, Knoxville, TN, USA

## Abstract

**Background:**

Cost is a commonly reported barrier to healthy eating. This is a secondary research analysis designed to examine the food expenditures of young adults on a university campus following the United States Department of Agriculture (USDA) MyPlate guidelines for fruits and vegetables.

**Methods:**

Meal receipts and dietary intake were recorded weekly. Anthropometrics and clinical assessments were recorded before intervention. Researchers rated compliance based on the participant's dietary food log, receipt matching, food pictures, and reports during weekly 1-hour consultations.

**Results:**

Fifty-three young adults (18–30 years old) at-risk of, or diagnosed with, metabolic syndrome (MetS) were enrolled in the study, with 10 excluded (*n* = 43) from analyses due to enrollment in a fixed cost university campus dining meal plan. A two sample *t*-test assessed differences in food costs and regression analysis determined associations between food cost and diet compliance while controlling for confounding factors of age, sex, and body mass index (BMI). Diet compliant subjects (*n* = 38) had higher weekly food cost at $95.73 compared to noncompliant subjects (*n* = 5) who spent $66.24 (*p*=0.01). A regression analysis controlling for age, sex, BMI, and geographical region also indicated cost differences based on diet compliance (*p* < 0.0001).

**Conclusion:**

Results indicate an ∼$29.00 per week increase in food cost when eating the recommended amount of fruit and vegetables. These findings can contribute to research incentive design, program planning cost, and determining effective interventions to improve diet in this population.

## 1. Background

Diet quality and weight status are modifiable factors that contribute to diet-related chronic diseases including cardiovascular disease (CVD), type 2 diabetes, and some cancers [1, 2]. Primary (preventing the onset of disease) and secondary (detecting disease in earliest stages) prevention of these diseases can be influenced by adopting healthy eating behavior practices as young adults. However, many young adults do not practice healthy eating [3]. A systematic review of diet quality in 187 countries found young adults aged 20–29 had lower dietary quality (44 points) than older adults (51 points) based on the Healthy Eating Index which is a validated scoring system rated out of 100 points [4]. The United States Departments of Health and Human Services (DHHS) and United States Department of Agriculture (USDA) Dietary Guidelines recommend Americans fill half their plate with fruits and vegetables at each meal (4-5 cups daily) [5]. However, only 12.3% of adults older than 20 years of age meet the recommended goals for fruit and vegetable intake [6]. For most young adults, the amount of fruit and vegetable intake is also suboptimal for the prevention of chronic diseases of adulthood [7].

Many of the benefits of healthy eating and maintaining a healthy weight are known. However, the continued rise in weight [3, 8] and decrease in food quality [9] indicate there are barriers to adopting healthier behaviors. Young adulthood is a time of self-definition, where individuals establish and practice healthy habits. These habits impact their weight status as they gain more independence in economic and dietary practices [10]. Key influences of dietary intake include current and past social environment, cost, preparation, purchase, and storage of food, knowledge, and motivation [3].

This study focuses on monetary costs associated with increasing intake of dietary fruit and vegetables in the context of an 8-week, personalized, and diet education program. Populations in the United Kingdom [11], Holland [12], and the United States [13, 14] report food cost to be a major barrier to buying healthy foods. Understanding the food cost of a healthy diet is important because even with an effective education program, access to healthy foods, and motivation, people may not make changes which can positively influence their health if they believe they cannot afford the food. Increasing cost is known to influence choice and behavior (e.g.,cigarettes and alcohol, where increasing costs, such as taxes decrease consumption [15]). In this study, we evaluate food costs associated with a positive dietary change.

The cost of a healthy dietary pattern (increased in fruits, vegetables, and lean protein) has been found to have an increased cost of $1.50 per day in the United States with healthier meat/protein options, contributing to the largest price difference [16]. Another study completed in Europe found there was an 18% increase in cost when the diet consisted of all five food groups instead of just two to three [17]. Mulik and Haynes-Maslow used the most current publicly available data from the USDA to analyze the price of the MyPlate's dietary guidelines for all food groups. Their findings indicate that men and women (19–30 years old) have an increased cost of food with no significant difference in the type of fruit or vegetable (fresh, canned, or frozen), with an average increase of $3.82 in women and $4.25 in men [18].

This increased cost can be an important consideration, especially in the young adult and college population. Thirty-two percent of college students report finances are traumatic or very difficult to handle [19]. Increased financial stress, higher cost of a healthy diet, and the young adult time period being understudied [7] indicate food cost for this age group is an area which needs further investigation.

This study was a secondary outcome analysis to a larger study to determine cardiovascular and gut microbiome changes in individuals after an 8-week dietary intervention which focused on increasing fruits and vegetables. The main objective of this prospective analysis was to determine the amount of money spent by young adults following the U.S. Dietary Guidelines (half of the plate consisting of fruits and vegetables). It was hypothesized that participants following the recommended diet would spend more money on food, compared to those who were not compliant with the diet. An increased food cost was determined because participants would be increasing fruit and vegetable intake which would take place of some of the cheaper, less healthy convenience food items which are frequently eaten by this group.

## 2. Methods

An 8-week diet intervention study was conducted with 53 young adults from West Virginia University (WVU) in two different cohorts, the spring of 2015 and the fall of 2016, investigating increased fruit and vegetable intake on clinical and metabolomic outcomes [20]. Recruitment occurred through word of mouth, flyers posted around campus, announcements in classrooms, and emails to the student body. To be eligible, participants had to be between the ages of 18–30, and be at-risk of, or diagnosed, with metabolic syndrome (MetS). “At-risk of MetS” was defined as 3 or more of the following risk factors: any of the 5 MetS risk factors in addition to BMI (>25 for men or women), personal or family history of CVD, diabetes (type 1, type 2, or gestational), or abnormal lipids, race/ethnicity, low physical activity, increased sedentary time, poor nutritional quality, current smoker, or excessive alcohol intake [21]. The guidelines set forth by the National Cholesterol Education Program Adult Treatment Panel III were used to diagnosis MetS. Individuals with three of the five following criteria were defined with MetS: waist circumference >102 cm (men) and >88 cm (women); serum triglycerides >150 mg/dl; serum HDL <40 mg/dl (men) and <50 mg/dl (women); blood pressure ≥130/85 mm Hg; and fasting blood glucose ≥100 mg/dl [22]. This was determined through in-person anthropometric and blood measurements. Exclusions included a diagnosis or treatment of a serious mental or behavioral disorder within the past year and pregnancy. Students eating the campus meal plan were removed from this analysis since they did not have out-of-pocket costs for much of their food consumption. Approval was obtained from the WVU Institutional Review Board, and informed consent was collected from each subject prior to enrollment in the study.

### 2.1. Outcome Measures

Demographic information was collected during the health assessment. Geographic region was defined as if the participant identified as being from an Appalachian (encompassing all of West Virginia and parts of 12 other states along the Appalachian Mountains), or non-Appalachian area. Clinical and nutrition history was obtained to assess risk and/or diagnosis of MetS. Weight was measured when participants were minimally clothed, without shoes using digital scales (SECA 874) and recorded to the nearest 0.1 kg. Height was measured in a standing position without shoes using a stadiometer (SECA 213). Body mass index (BMI) was calculated and expressed in kg/m^2^. Waist circumference was measured at the narrowest point, and hip circumference was measured at the maximum point over light clothing using a Gulick tape meter. Height and waist circumference measurements were recorded to the nearest 0.1 cm after being taken twice and averaged for analysis.

Diet compliance was determined through subjective and objective measures to eliminate bias throughout the 8-week intervention. Compliance with diet and assessment of food expenditures were determined through food pictures (generally taken on their phone and emailed or shown to researcher during counseling session), participant's dietary food log (matched to food shopping receipts), food pictures (to determine portion sizes), and weekly 1-hour consultation with a trained researcher. Cost of all liquid and solid food and alcohol were determined through food receipts collected from participants every week. Costs were recorded and labeled according to location the food was purchased. Receipts were retrieved from participants for grocery store, restaurant, vending machine, cafe, and any other food establishment purchases. Participants recorded the cost of food in individual food logs when receipts were unavailable.

### 2.2. Study Procedures

Each participant was instructed to follow a calorie intake (based on the U.S. Dietary Guidelines for Americans) to maintain weight calculated using their current weight, age, and physical activity status; they were asked to maintain their current activity level. Participants were educated on this diet during a two-hour education session prior to the beginning of the study which included food intake expectations, research protocol, healthy eating on a budget, menu ideas, recipes, and sample grocery lists. All subjects were asked to consume a diet consisting of 4-5 servings (cup/ounce equivalents) fruit and vegetables and not change their dietary supplement intake. As part of the main project protocol, participants in the first cohort were also randomly assigned to follow the increased fruit and vegetable diet only or to additionally follow low-refined carbohydrate or low-fat recommendations. All participants within these different groups were evaluated in this paper. Each participant had their USDA MyPlate food group recommendations calculated using the computer software program Nutritionist ProTM (Axxya Systems LLC, Redmond, WA). Participants were provided with kitchen tools (measuring cups and spoons, Tupperware containers, knives, etc.) to facilitate food preparation at home, and financially compensated throughout the 8-week study for a total of $250, which was split into a smaller amount to give at their weekly consultations.

During the weekly consultation, participants also reviewed their daily food log with researchers that were analyzed using Nutritionist Pro. At this time, researchers would use motivational interviewing techniques (all had completed a two-day training) to facilitate behavior change [23, 24] and develop strategies to reach personalized goals. The weekly dietary reports were used as markers to gauge participant improvement and dietary intervention compliance. Dietary compliance was defined as maintaining the diet intervention guidelines 75% of the time during 6 weeks of the intervention, as determined by the researcher.

### 2.3. Statistical Analysis

Food costs were analyzed with the Stata 14 software system [25]. Two sample *t*-tests with unequal variances were used to determine differences in food costs between compliant and noncompliant dietary intake status. The unequal variances are used to account for the uneven compliant and noncompliant groups [26]. Results were considered significant if the two-tailed *p* value was ≤0.05. It should be noted that this is a secondary analysis to a larger research study, and thus, the sample size was not powered for this analysis.

## 3. Results

Ten participants from the primary study group were excluded from this analysis (*n* = 43) because of enrollment in a campus dining plan which is based on fixed quarterly cost. This study sample was 60% female, and most individuals were diet compliant (88%). Participant living arrangements varied though all reported living outside of the home where they grew up. Demographic information is presented in Table 1, and baseline clinical measurements are included in Table 2. Among participants, there was an even distribution of BMI categories (normal weight = 15, overweight = 10 and 8, and morbidly obese = 10). A detailed analysis of the dietary intake associated with the larger study has been previously reported [20, 27]. By the end of the 8-week study, participants had demonstrated improvements in fruit and vegetable consumption, fiber intake, and a decrease in empty calorie intake with no supplement intake included in the analysis.

**Figure 1:**
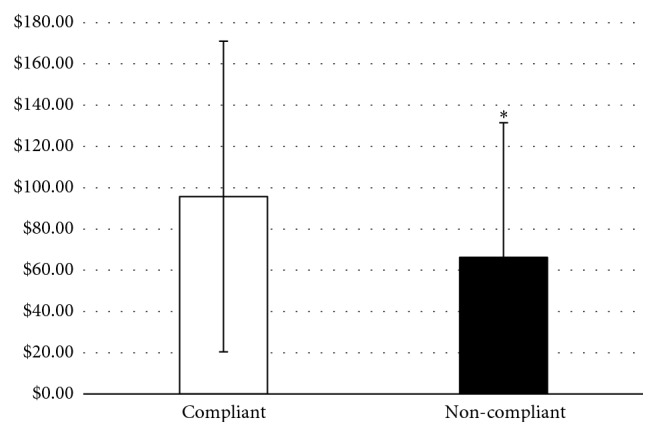
Mean ± SD cost (dollars) of compliant (*n* = 38) and noncompliant (*n* = 5) participants during the 8-week diet intervention (*p*=0.02).

Compliant individuals, on average, spent $95.73 ± $75.33 per week compared to noncompliant individuals spending $66.24 ± $65.31 per week.  Figure 1 shows the average weekly spending of these two groups throughout the 8-week study. Compliant participants spent more on food compared with noncompliant participants (*p*=0.02). Spending differences between compliant and noncompliant groups remained after controlling for age, sex, BMI, and region (*p* < 0.0001).

## 4. Discussion

Many studies report cost as a barrier which deters some people from buying healthy foods [11–14]. The principal finding of the present study was that participants who were compliant with the MyPlate diet spent, on average, $29 more per week on food (∼$4 a day) than noncompliant individuals. This finding correlates with prior studies, indicating the higher costs of a healthy diet. A meta-analysis of 27 studies across 10 countries found a healthy diet cost $1.48 more a day than less healthy options [16]. However, a more recent analysis of the cost of the MyPlate recommendations for individuals in this age group did have a similar price increase to the current study ($3-4/day depending on gender and whether fresh, frozen, or canned fruits and vegetables were bought) [18]. The large standard deviations in some of the results may be due to participants not being required to buy groceries every week. Instead, on some weeks, participants' food cost would be very low since they had bought enough food to last more than one week. Below there will be an exploration of possible reasons for the food cost differences experienced in the groups including the cost of fresh food, young age of the subjects, and geographic location.

In this study, participants bought a variety of food options from all five food groups at restaurants, convenience, and grocery stores. The findings in this study are consistent with the patterns of increased food cost when on an isocaloric diet and asked to increase nutrient density (higher intake of fruits and vegetables). Diets lower in cost were associated with lower consumption of vegetables, fruits, whole grains, and seafood [28]. Another study of 837 French adults separated food cost by food group and concluded that individuals eating more fruits and vegetables in their diet incurred a higher food cost [29]. Another way to look at nutritional quality is using a measure of nutrient density. Foods with a higher nutrient content or density are frequently higher in cost compared to less healthy, calorie-dense options [30]. Further qualitative exploration was completed to analyze group differences in spending habits for this sample size. Noncompliant individuals purchased a low quantity of fruits and vegetables and a high amount of grains and fats from convenience foods. Compliant individuals purchased more fruits and vegetables and a larger variety of food from different food groups. Compliant individuals that spent more money on food tended to purchase more seafood and meat and had a higher frequency of eating away from home compared to compliant individuals that spent less money on food purchases.

In understanding the results of this study, it is important to consider the unique experiences, education, and financial situations of young adulthood. Young adults and university students commonly do not have a traditional job and income stream and have been found to make suboptimal financial decisions [31]. When stratifying diet cost by age group, young adults (20–29 years of age) spent the least and had the lowest diet quality compared to any other age groups [28]. The survey of young adults (18–38 years old) in the Bogalusa Heart Study found those with lower income ate fewer fruits and vegetables, more fats and sweetened beverages, and statistically higher amounts of burgers and sandwiches [32]. The income of young adults as well as their inclination for ready-to-eat, processed, frozen, or canned foods for convenience may be a significant variable for young adults in college to eat a healthy diet [32–34].

To increase fruit and vegetable consumption, studies have employed educational interventions, public campaigns, and price reductions. The Supermarket Healthy Eating for Life Trial conducted a randomized controlled trial over three months to determine if a price reduction of fruits and vegetables would result in increased fruit and vegetable consumption. A 20% reduction in price resulted in a 35% increase in fruit and a 15% increase in vegetable purchases. The behavior was not maintained six months after the intervention when food returned to their original cost [35]. A recent study developed a model to compare the effectiveness of a multimedia campaign or price decrease to increase fruit and vegetable consumption using current diet trends, national databases, and other studies to determine projected change. This study demonstrated that media and financial interventions increased fruit and vegetable intake, although the effect of the price reduction was more powerful and sustainable [36]. This supports the role of price incentive or reduced cost as a factor in food purchasing decisions. Our study provided research participation incentives ($250 total/eight weeks) that may have been used to offset participants' food costs to enable them to buy healthier options, though the financial incentive could have been spent on other items as well.

What an individual chooses to eat is multifactorial and has other determinants besides cost. Other factors can include taste, convenience, interest in health and nutrition, familiarity of the food, cooking skills, and mental health [37, 38]. NHANES data determined taste was the most important factor in food decisions, followed by nutrition, cost, and convenience [39]. Carlson and Frazao further explored reasons for food decisions. In this study, it concluded that higher income individuals may spend more money on food, but their diet was not necessarily healthier than low-income individuals [40]. This indicated that spending more money on food did not guarantee the food being purchased was healthy. So, aside from just financially incentivizing healthy food purchases, it is important to incorporate education on nutrition as well as how to cook and prepare healthy foods that are palatable to the individual. In the population being studied here, there were compliant individuals who were able to spend less money, so with more education, it may be possible to teach the participants how to have a healthy diet with lower cost.

The current study being examined used the combined education and financial incentive component to encourage healthy eating. Another point which should be explored is the perceptions that all healthy food is expensive. For example, the cost of the Mediterranean diet, which is viewed as being healthful, is perceived to cost more. However, some components of the diet cost less (e.g., certain vegetables, beans, legumes, grains, nuts, and some dairy products) and can replace the more expensive items [41]. The fact that healthier diets can cost less given different food-related decisions is a phenomenon called nutrition resilience. This indicates healthy diets can be maintained at a lower cost, given optimal decision-making and knowledge of how to eat healthy on a budget [42]. However, it may take more time than the current study (8 weeks) for individuals to be educated on, explore, and put into practice buying and eating food items that are healthy and affordable.

This study has limitations. First, there was a small number of individuals in the noncompliant group. This could have influenced the resultant higher cost for diet compliant individuals as well as the difference in the cost of healthy eating by the BMI category. To accommodate the uneven group sizes, a *t*-test with unequal variances was used in the statistical analysis [26]. Second, the amount of money spent on the different food groups was not determined in this study, though dietary intake reveals participants' fruit and vegetable intake increased. This study was intended to use the basic statistical analysis to compare compliant fruit and vegetable intake and diet cost and thus did not have enough information to accurately analyze the types of food participants were purchasing. Third, income was not obtained which may have also helped to see if that played a role in the amount of money spent on food. To overcome these limitations, further studies should more thoroughly define the locations of the purchased food, as well as the food groups purchased, and include larger sample sizes of young adults from several different geographic regions. This would help to develop further explanations for these groups' different food-spending habits.

Further qualitative exploration was completed to analyze group differences in spending habits for this sample size. In general, students purchased a variety of food options from all five food groups at restaurants, convenience, and grocery stores. Noncompliant individuals purchased a low quantity of fruits and vegetables and a high amount of grains and fats from convenience foods. Compliant individuals purchased more fruits and vegetables and a larger variety of food from different food groups. Compliant individuals that spent more money on food tended to purchase more seafood and meat and had a higher frequency of eating away from home compared to compliant individuals that spent less money on food purchases.

## 5. Conclusions

This study contributes new data on the costs of implementing the USDA MyPlate guidelines for young adults living in a university setting. This age group spent $29 more per week, on average, when complying with the national health guidelines. These findings can contribute to research incentive design and program planning cost and determining effective interventions to improve diet in this population. Future research regarding food costs is needed with this age group as well as an expanded analysis to include what food groups or choices are contributing to the food cost. Additional knowledge can contribute to education and public health interventions in this population to increase the affordability of healthy foods and give the education needed by this age group to improve budgeting and food preparation skills to be able to use the healthy foods in a way that is palatable to their tastes and lifestyle.

## Figures and Tables

**Table 1:** Demographic characteristics of all included participants at baseline (*n* = 43).

	Included^a^, *n* (%)
Age (*x* ± SD)	22.2 ± 3.4
Sex (male)	17 (39.5)
Race/ethnicity	
White	29 (67.4)
African American	5 (11.6)
Asian	4 (9.3)
Hispanic	3 (7)
American Indian	1 (2.3)
Middle East	1 (2.3)
From Appalachia	25 (58.1)

^a^Ten participants were excluded from this analysis due to enrollment in a campus dining meal plan.

**Table 2:** Baseline clinical measurements (*n* = 43).

Variable	Mean ± SD
Waist circumference (cm)	90.8 ± 15.6
Body mass index (kg/m^2^)	30.1 ± 7.3
Systolic blood pressure (mm/Hg)	116 ± 14
Diastolic blood pressure (mm/Hg)	65 ± 10
Serum glucose (mg/dL)	88 ± 7
High-density lipoprotein (mg/dL)	53 ± 13
Triglycerides (mg/dL)	102 ± 48

## Data Availability

The datasets used and/or analyzed during the current study are available from the corresponding author on reasonable request.

## References

[1] Murray Christopher J. L. (2013). The state of US health, 1990–2010. *Journal of the American Medical Association*.

[2] Holben D. H., Zurmehly A., Jackson L., Holcomb J. P. (2009). Food insecurity is associated with increased diabetes risk, obesity, and poorer perceived diet and health among women in rural Appalachian Ohio. *Journal of the American Dietetic Association*.

[3] Munt A. E., Partridge S. R., Allman-Farinelli M. (2017). The barriers and enablers of healthy eating among young adults: a missing piece of the obesity puzzle: a scoping review. *Obesity Reviews*.

[4] Imamura F., Micha R., Khatibzadeh S. (2015). Dietary quality among men and women in 187 countries in 1990 and 2010: a systematic assessment. *The Lancet Global Health*.

[5] United States Department of Health and Human Services (2015). *2015–2020 Dietary Guidelines for Americans*.

[6] Go A. S., Mozaffarian D., Roger V. L. (2014). Heart disease and stroke statistics—2014 update: a report from the American Heart Association. *Circulation*.

[7] Lipsky L. M., Nansel T. R., Haynie D. L. (2017). Diet quality of US adolescents during the transition to adulthood: changes and predictors. *American Journal of Clinical Nutrition*.

[8] Allman-Farinelli M. (2015). Nutrition promotion to prevent obesity in young adults. *Healthcare*.

[9] Glendinning G., Alaunyte I., Amirabdollahian F. (2015). An investigation into gender variation in the nutritional status of young adults. *Proceedings of the Nutrition Society*.

[10] Nelson M. C., Story M., Larson N. I., Neumark-Sztainer D., Lytle L. A. (2008). Emerging adulthood and college-aged youth: an overlooked age for weight-related behavior change. *Obesity*.

[11] Hampson S. E., Martin J., Jorgensen J., Barker M. (2009). A social marketing approach to improving the nutrition of low-income women and children: an initial focus group study. *Public Health Nutrition*.

[12] Waterlander W. E., De Mul A., Schuit A. J., Seidell J. C., Steenhuis I. H. (2010). Perceptions on the use of pricing strategies to stimulate healthy eating among residents of deprived neighbourhoods: a focus group study. *International Journal of Behavioral Nutrition and Physical Activity*.

[13] Hill S. E., Baskett K., Bradshaw H. K., Prokosch M. L., DelPriore D. J., Rodeheffer C. D. (2016). Tempting foods and the affordability axiom: food cues change beliefs about the costs of healthy eating. *Appetite*.

[14] Dammann K. W., Smith C. (2009). Factors affecting low-income women’s food choices and the perceived impact of dietary intake and socioeconomic status on their health and weight. *Journal of Nutrition Education and Behavior*.

[15] Chaloupka F. J., Grossman M., Saffer H. (2002). The effects of price on alcohol consumption and alcohol-related problems. *Alcohol Research and Health*.

[16] Rao M., Afshin A., Singh G., Mozaffarian D. (2013). Do healthier foods and diet patterns cost more than less healthy options? A systematic review and meta-analysis. *BMJ Open*.

[17] Conklin A. I., Monsivais P., Khaw K.-T., Wareham N. J., Forouhi N. G. (2016). Dietary diversity, diet cost, and incidence of type 2 diabetes in the United Kingdom: a prospective cohort study. *PLoS Medicine*.

[18] Mulik K., Haynes-Maslow L. (2017). The affordability of MyPlate: an analysis of SNAP benefits and the actual cost of eating according to the dietary guidelines. *Journal of Nutrition Education and Behavior*.

[19] Association ACH: American College Health Association-National College Health Assessment II (2017). *Reference Group Executive Summary Fall 2016*.

[20] Mathews A. T., Famodu O. A., Olfert M. D. (2017). Efficacy of nutritional interventions to lower circulating ceramides in young adults: FRUVEDomic pilot study. *Physiological Reports*.

[21] Olfert M., Famodu O., Clark R. (2017). Development of an ’at risk for metabolic syndrome’ score. *Journal of Nutrition Education and Behavior*.

[22] Miccoli R., Bianchi C., Odoguardi L. (2005). Prevalence of the metabolic syndrome among Italian adults according to ATP III definition. *Nutrition, Metabolism and Cardiovascular Diseases*.

[23] Miller W. R., Rollnick S. (2012). *Motivational Interviewing: Helping People Change*.

[24] Armstrong M., Mottershead T., Ronksley P., Sigal R., Campbell T., Hemmelgarn B. (2011). Motivational interviewing to improve weight loss in overweight and/or obese patients: a systematic review and meta-analysis of randomized controlled trials. *Obesity Reviews*.

[25] StataCorp (2015). *Stata Statistical Software: Release 14*.

[26] Ruxton G. D. (2006). The unequal variance *t*-test is an underused alternative to Student’s *t*-test and the Mann–Whitney *U* test. *Behavioral Ecology*.

[27] Clark R. L., Famodu O. A., Barr M. L. (2017). Fruit and vegetable diet intervention in young adults with metabolic syndrome: FRUVEDomics pilot study. *FASEB Journal*.

[28] Rehm C. D., Monsivais P., Drewnowski A. (2015). Relation between diet cost and Healthy Eating Index 2010 scores among adults in the United States 2007–2010. *Preventive Medicine*.

[29] Drewnowski A., Darmon N., Briend A. (2004). Replacing fats and sweets with vegetables and fruits-A question of cost. *American Journal of Public Health*.

[30] Darmon N., Briend A., Drewnowski A. (2004). Energy-dense diets are associated with lower diet costs: a community study of French adults. *Public Health Nutrition*.

[31] Letkiewicz J. C. (2012). *Self-Control, Financial Literacy, and the Financial Behaviors of Young Adults*.

[32] Deshmukh-Taskar P., Nicklas T. A., Yang S.-J., Berenson G. S. (2007). Does food group consumption vary by differences in socioeconomic, demographic, and lifestyle factors in young adults? The Bogalusa Heart study. *Journal of the American Dietetic Association*.

[33] Drewnowski A. (2004). Obesity and the food environment. *American Journal of Preventive Medicine*.

[34] French S. A. (2003). Pricing effects on food choices. *Journal of Nutrition*.

[35] Ball K., McNaughton S. A., Le H. N. (2015). Influence of price discounts and skill-building strategies on purchase and consumption of healthy food and beverages: outcomes of the Supermarket Healthy Eating for Life randomized controlled trial. *American Journal of Clinical Nutrition*.

[36] Pearson-Stuttard J., Bandosz P., Rehm C. D. (2017). Comparing effectiveness of mass media campaigns with price reductions targeting fruit and vegetable intake on US cardiovascular disease mortality and race disparities. *American Journal of Clinical Nutrition*.

[37] Todd J. E. (2014). *Changes in Eating Patterns and Diet Quality Among Working-Age Adults, 2005–2010*.

[38] Deaton A., Muellbauer J. (1980). *Economics and Consumer Behavior*.

[39] Aggarwal A., Rehm C. D., Monsivais P., Drewnowski A. (2016). Importance of taste, nutrition, cost and convenience in relation to diet quality: evidence of nutrition resilience among US adults using National Health and Nutrition Examination Survey (NHANES) 2007–2010. *Preventive Medicine*.

[40] Carlson A., Frazão E. (2014). Food costs, diet quality and energy balance in the United States. *Physiology & Behavior*.

[41] Drewnowski A., Eichelsdoerfer P. (2009). The Mediterranean diet: does it have to cost more?. *Public Health Nutrition*.

[42] Drewnowski A., Kawachi I. (2015). Diets and health: how food decisions are shaped by biology, economics, geography, and social interactions. *Big Data*.

